# Physico-Chemical Properties and Chemical Analysis of Wildflower Honey Before and After the Addition of Spirulina (*Arthrospira platensis*)

**DOI:** 10.3390/molecules29184373

**Published:** 2024-09-14

**Authors:** Cosimo Taiti, Lara Costantini, Diego Comparini, Nicolò Merendino, Stefania Garzoli

**Affiliations:** 1 Department of Agriculture, Food, Environment and Forestry (DAGRI), University of Florence, 50144 Florence, Italy; cosimo.taiti@unifi.it (C.T.); diego.comparini@unifi.it (D.C.); 2 Department of Ecological and Biological Sciences (DEB), Tuscia University, Largo Dell’Università Snc, 01100 Viterbo, Italy; lara.cost@unitus.it (L.C.); merendin@unitus.it (N.M.); 3 Department of Drug Chemistry and Technology, Sapienza University, 00185 Rome, Italy

**Keywords:** chemical analyses, volatile compounds, sugars, HMF, proximate composition

## Abstract

In this study, in order to verify the effects due to the addition of spirulina (*Arthrospira platensis*) in a food product, a wildflower honey was analyzed in terms of chemical composition, physicochemical properties and antioxidant activity before and after the addition of the spirulina. HS-SPME/GC–MS and HPLC/UV were applied to carry out the chemical analyses. The obtained results demonstrated that the volatile profile and also the sugar content were significantly influenced by the addition of spirulina, showing significant qualitative and quantitative differences compared to honey without spirulina. The increase in HMF in honey added with spirulina was significant, demonstrating that its presence could accelerate the Maillard reaction. Electrical conductivity measured by using a conductometer was also increased while the moisture content was reduced in honey enriched with spirulina. Instead, the pH value was similar between the two samples. On the other hand, honey fortification with spirulina determined a significant increase of 12.5% in the total phenolic content (TPC), and a 56.25% increase in protein content. Further, the total antioxidant capacity (TAC) was also evaluated and a significant increase was determined as a result of the addition of spirulina. In conclusion, honey enriched with *A. platensis* was found to be characterized by a high pool of bioactive metabolites as well as significant changes in almost all the measurements performed.

## 1. Introduction

Honey is a sweet, viscous-looking natural food with a certain aromatic character consumed throughout the world. Honey is characterized by a high content of sugars and by amino acids, organic acids, vitamins and also aromatic substances. These constituents are responsible for the beneficial properties of honey, including antibacterial and antioxidant properties [1]. The composition of honey as well as its aroma and flavor depend on various environmental factors such as geographical position and atmospheric events and obviously on the species of bees involved in the production; to these factors we then add the production phase which can still influence its characteristics [2,3].

In general, honey is a food that can undergo changes during storage due to oxidation reactions or heat treatment [4]. In fact, when honey is heated or stored for a long time, the formation of furan derivatives occurs [5] such as furfural, which derives from pentoses, and 5-hydroxymethylfurfural (5-HMF), which is derived from hexoses such as glucose and fructose [4]. These compounds are the classic degradation products of sugar in an acidic environment and caramelization. 5-HMF as a product of the Maillard reaction [3] is, therefore, an indicator of the quality of the honey itself as, if present at high concentrations, it can be toxic [6,7,8].

The sugars present in honey are mainly monosaccharides followed by a lower percentage of disaccharides [9]. The concentration of the monosaccharides fructose and glucose and also their ratio are useful indicators for the classification of honey [10]. Generally, fructose is superior to glucose except in some cases [2]. However, the sugars in honey can change and/or vary their concentration during the storage phase [11]. Other substances that form over time from the dehydration of sugars can also induce physical changes in honey, such as a darker color and a change in flavor [8].

The contents of organic acids in honey were also reported which are linked to the color and flavor of the honey and obviously to its acidity [12]. Gluconic acid and citric acid are generally the most abundant. To these are also added levulinic and formic acids which can derive from 5-HMF and whose concentrations are used as a reliable parameter to differentiate floral honey from honeydew [12]. The presence of 5-HMF in the honey can inevitably contribute to increasing the acidic character of honey with the passage of time [13].

The volatile fraction in honey is very rich and the volatile compounds can have different origins. In fact, they may result from the transfer of volatile compounds from plants or by the presence of microorganisms [6,14]. Generally, monoterpenes and sesquiterpenes are present in honey at very low concentrations [15]. In any case, they are responsible for the flavor which can vary to the point of even becoming rancid [6]. In contrast, alcohols such as 3-methyl-3-butene-1-ol and 2-methyl-2-buten-1-ol, can give freshness to honey [7]. Previous studies reveal that the volatile profile varies during honey storage [4]. Hence the evaluation of the volatile component of honey should be performed over time to verify the reduction or loss of some components. In summary, all the aspects listed above are important in characterizing honey as they are responsible for the olfactory characteristics, taste, flavor as well as the quality of the final product.

Following the increasingly numerous requests from consumers to try food products with different flavors and tastes, the food industry is increasingly committed to producing functional foods thanks to the addition of substances that can not only increase their palatability but also improve their supply of nutrients.

Among the foods, honey, which is already a natural product with a high nutritional profile, can be enriched with a limited number of ingredients including spirulina algae to increase its benefits for human health. Spirulina (*A. platensis*), is a blue-green algae and a non-toxic species of cyanobacteria and a great nutritional source [16]. Thanks to its rich nutrient content, many biological activities were attributed to it including antioxidant, anti-inflammatory and also reparative activities in the case of metabolic disorders [17].

In the present work, with the aim to better comprehend the effects resulting from the addition of *A. platensis*, a comparative study between an Italian wildflower honey with and without organic spirulina produced in Tuscany, was conducted. In particular, an evaluation of the chemical–physical properties and of the VOCs, sugar, protein and total phenolic content and the antioxidant capacity was carried out applying multiple analytical methods.

## 2. Results and Discussion

### 2.1. VOCs from Honey

By SPME-chromatographic analyses carried out on the untreated wildflower honey and wildflower honey with spirulina, 24 volatile components were detected and identified (Table 1). Relevant qualitative differences between the two samples were found. In detail, hotrienol, an aliphatic alcohol, was detected as the main compound in wildflower honey (36.5%) while in honey with spirulina this compound covered only 2.1% of the total. Furthermore, no monoterpenes present in the control honey were detected in the latter (12.8%). Generally, honey is a very poor food in terms of lipid and fatty acid content. According to our results, the addition of spirulina led to an increase in the fatty acid content detected in the headspace of the samples (80.9% versus 33.9%), in particular, the percentage of palmitic acid rose to 24.3%, that of elaidic acid to 40.7% and that of oleic acid to 15.9%. These data are in agreement with Guldas et al. [17], who reported that the presence of spirulina in honey caused a significant increase in the overall fatty acid content.

Our results also show that the honey with spirulina contained a certain number of hydrocarbons such as pentadecane (0.6%), hexadecane (0.8%) and heptadecane (9.8%), which were not detected in the control honey.

Several factors such as heat treatment and storage conditions can contribute to the presence of volatile compounds in honey. In particular, some compounds can undergo oxidation or degradation [18]. In this regard, volatile extraction techniques can also lead to the formation of some compounds unrelated to the origins of honey.

In order to provide a description of the volatile profile of the honey samples investigated as realistically as possible, in this study, we performed the HS-SPME technique. The main advantages of headspace sampling are its operational simplicity, the non-use of organic solvents and no manipulation of the sample thus significantly reducing the possible alterations induced by classic extraction processes [15,19]. In recent years, an ever-increasing number of works on the volatile composition of different monofloral kinds of honey have been published, highlighting the interest in this natural product [20,21]. There are currently around 600 volatile compounds identified in honey of different origins even if their concentration is very low [18]. However, to the best of our knowledge, this is the first work that reports on the effect of adding spirulina to honey on the volatile fraction.

### 2.2. GC–MS Analysis of Dried Honey Extracts

In addition to the determination of sugars, the analysis conducted on the methanolic extracts from dry honey allowed the identification of other molecules belonging to different chemical classes such as carboxylic acids, alcohols and traces of terpenes. A total of 38 different compounds were detected and identified (Table 2). The results highlighted how the sugar fraction was the predominated one. Qualitative and quantitative differences between the two samples were evident; in particular, the carbohydrates D-fructose, β-D-glucopyranose and myo-inositol were significantly higher in wildflower honey (21.1%; 25.6%; 10.6%) than in honey with the addition of spirulina (13.3%; 3.5%; 0.1%). In contrast, D-fructofuranose (29.7%) and D-ribofuranose (15.7%) were prevalent in honey with spirulina. Generally, in almost all types of honey, fructose is proportionally higher than glucose except in some honey such as dandelion honey (*Taraxacum officinale*), where the ratio is reversed thus leading to rapid crystallization [2]. The obtained data showed that the addition of spirulina resulted in a decrease in the total carbohydrate content compared to the control (89.5% versus 98.2%, respectively). Previous works showed similar differences in the carbohydrate content of some foods following the addition of spirulina. For instance, when enriching pasta with spirulina, a proportional decrease in carbohydrate content was reported [22]. Tańska et al. [23] also reported a decrease in carbohydrate content in corn extrudates enriched with spirulina.

Another substantial difference concerned the presence of alcohols and carboxylic acids found in honey with spirulina; only some of which were present, in traces, in the control honey. Among carboxylic acids, 3-methyl-2-furoic acid (2.4%) was detected as the most abundant one. Formic acids can derive from 5-HMF following subsequent reactions and this leads to an increase in the concentration of free acidity in honey. Furthermore, the presence of alcohols in honey with spirulina could be related to the greater presence of acids detected in this honey. Indeed, it was reported that the acidity of honey increases during fermentation as the sugars and alcohols in honey are transformed into acids by the action of honey yeasts [13]. Among other things, organic acids are also related to certain intrinsic properties such as color and flavor [24].

### 2.3. Physico-Chemical Results

Table 3 provides a summary of the results of the physico-chemical composition of the control honey and the honey with added spirulina. The physico-chemical analysis revealed some differences among the samples analyzed. On one hand, the degree of Brix and the pH value are similar between the samples studied, with no significant differences observed (Table 3), in accordance with the results proposed by Guldas et al. [17]. On the other hand, adding spirulina to honey affects the moisture content, electrical conductivity (EC), and hydroxymethylfurfural (HMF) content of the final product. The moisture content in both samples studied is under the maximum limit of 20% defined by the EU honey legislation. Spirulina, being a powdered algae, likely absorbed a certain amount of moisture from the honey, reducing the overall moisture content as reported in Table 3. Basuny et al. [25] also observed a reduction in moisture content in a yogurt drink to which spirulina powder was added. The moisture content of honey is crucial for its quality, stability, resistance to spoilage, and shelf life. Higher moisture content increases the risk of fermentation during storage, while lower moisture levels extend the shelf life of honey.

In addition, the addition of spirulina seems to affect the EC content of wildflower honey, which changes from an average value of 0.74 (+0.02) to 0.88 (0.02). The increase in EC could be due to the high quantity of mineral salts present in spirulina. Indeed, as reported by Janda-Milczarek and co-authors [26], spirulina powder contains mineral salts such as iron, potassium and magnesium. Since the HMF content derives from the degradation of fructose and is directly linked to the aging process of honey [27]. We tested if the addition of spirulina could affect this parameter. The HPLC quantification of HMF showed that the samples studied had an HMF content within the standard thresholds of 40 (mg/kg) as defined by legislation. The content of HMF increased significantly due to the enrichment of the honey with spirulina (*p* > 0.05) and changed from an average value of 2.40 (mg/kg) in the control honey to 8.13 (mg/kg) in the treated samples (Table 3). Thus, it seems that the addition of spirulina could accelerate the Maillard reaction (which is the major cause of HMF production) that occurs during the honey storage and increase the HMF content.

### 2.4. Total Phenolic Content (TPC), Total Antioxidant Capacity (TAC), and Protein Content Results

Here, for the first time, the contribution *spirulina platensis* powder at the TPC and TAC of honey was analyzed. The TPC analysis highlighted that the honey sample fortified with spirulina had significantly higher quantities of phenolic compounds. Indeed, adding spirulina powder determined a 12.5% increase in the TPC of the honey sample (Table 4). Furthermore, the presence of spirulina determined a significant increase in the TAC determined by the ABTS^•+^ method, but not by the less sensitive FRAP method. Indeed, ABTS^•+^ identified a 19.45% significant increase in the TAC of the honey fortified with spirulina powder. Although the FRAP analysis found a higher antioxidant capacity for the fortified honey sample, this failed to find a significant difference due to the high standard deviation. Anyway, it should be noted that direct fortification with spirulina platensis powder, rather than feeding bees with spirulina, for the first time determines a greater increase in both TAC and TPC in the experimental samples [17]. Indeed, Guldas and colleagues [17] did not find significant increases in the TPC of spirulina honey compared to the control, given the value of 0.139 mg GAE/g versus 0.99 mg GAE/g of the present samples (Table 4). Higher and significant values were also found for the TAC of wildflower honey with spirulina compared to the control (19.45% increase found by ABTS^•+^; Table 4) and compared to honey samples obtained by feeding bees with spirulina (2.29% increase found by Guldas and colleagues with the ABTS^•+^ method) [17]. Furthermore, here, for the first time, the protein enrichment of honey due to the addition of spirulina, was analyzed.

Considering the high protein content of the present spirulina samples, as determined by us previously (54.84%) [28], the honey fortification with spirulina resulted in a significant increase in the protein content of 56.25%.

## 3. Materials and Methods

### 3.1. Materials

Honey samples, commercial products, currently on sale, were purchased by Spirulina Becagli Farm, 58100 Grosseto, Italy. Biobacche Toscane, Grosseto, Italy, was the honey-producing company. Wildflower honey is made using a blend of nectars from different flowers and has a deep yellow color with orange reflections. Honey production is carried out by minimising product handling to ensure maximum integrity of the final product. Honey extraction and bottling are carried out without resorting to honey heating processes. The percentage of spirulina added to the multi-flower honey was 0.5% (dry powder). The spirulina-enriched honey came from the same production batch as the control wildflower honey. Spirulina Becagli Farm is also the home of the organic spirulina biomass production facility previously investigated in our recent work [28].

All chemicals were purchased from Sigma-Aldrich (St. Louis, MO, USA).

### 3.2. SPME Sampling

To describe the volatile chemical profile of the honey samples, SPME sampling technique was used. About 2 gr of each sample were placed inside a 7 mL glass vial with PTFE-coated silicone septum. To collect the volatiles from the headspace of the samples, a SPME device from Supelco (Bellefonte, PA, USA) with 1 cm fiber coated with 50/30 μm DVB/CAR/PDMS (divinylbenzene/carboxen/polydimethylsiloxane) was used. Before use, the fiber was conditioned at 270 °C for 30 min. After achieving equilibration, obtained by heating to a suitable temperature and time, the fiber was exposed to the headspace of the samples for 30 min at 60 °C to capture and concentrate the components. Lastly, the SPME fiber was inserted in the GC injector maintained at 250 °C in splitless mode for desorption of the compounds.

### 3.3. Extraction and Derivatization of Dried Honey Samples

To describe the non-volatile content, approximately 0.5 g of each sample was mixed with 20 mL of methanol and then ultrasound-assisted liquid–solid extraction was performed at 40 °C (sonication time of 10 min; fixed frequency of 50 Hz). The obtained extracts were dried over anhydrous sodium sulphate and then evaporated to dryness via a rotary evaporator. Approximately 2 milligrams of the dry residue obtained were derivatized with 100 μL of BSTFA and 200 μL of pyridine and heated at 70 °C for 1 h. The silyl derivatives were subjected to GC–MS analysis.

### 3.4. GC–MS Analysis

To investigate the headspace from honey samples, the analysis was carried out on Clarus 500 model Perkin Elmer (Waltham, MA, USA) gas chromatograph coupled with a mass spectrometer equipped with an FID (flame detector ionization). The chosen capillary column was a Varian Factor Four VF-1. The GC oven programmed temperature was set initially at 50 °C for 0.5 min then increased to 120 °C at 6°/min then ramped to 220 °C at 8 °C/min for 7 min then ramped at 15 °C/min to 260 °C min and finally held for 15 min. Helium was used as carrier gas at a constant rate of 1 mL/min (splitless mode). MS detection was performed with electron ionization (EI) at 70 eV operating in the full-scan acquisition mode in the *m*/*z* range 40–500 amu. The identification of compounds was performed by the comparison of the MS-fragmentation pattern of the analytes with those of pure components stored in the Wiley 2.2 and Nist 11 mass spectra libraries database. Further, the Linear Retention Indices (LRIs) were calculated using a series of alkane standards (C_8_–C_25_ *n*-alkanes). The obtained LRIs were compared with available retention data reported in the literature. The relative amounts of the components were expressed as percent peak area relative to total peak area without the use of an internal standard and any factor correction. The analysis was carried out in triplicate.

On the other side, to detect sugars and other non-volatile components, the same apparatus was used. In this case, the temperatures were programmed from 70 °C to 170 °C at 7°/min then ramped to 250° at 8 °C/min for 1 min then ramped at 8 °C/min to 300 °C min and finally held for 15 min. Helium was used as carrier gas at a constant rate of 1 mL/min (split 1:20). The full scan mode operated from 45 to 600 *m*/*z*. The identification of the components was based on the percentage of similarity plus comparison of mass spectra (MS) using software NIST 11 data library, with the percentage of total ion chromatograms (TIC%).

### 3.5. Physico-Chemical Analysis

For the analysis of pH, electrical conductivity, and hydroxymethylfurfural content, samples were prepared by a 1:5 dilution with ultrapure distilled water from a Millipore Milli-Q lab (Merck KGaA, Darmstadt, Germany) water system. Honey samples were thoroughly mixed to ensure homogeneity, after which a 5 g aliquot was taken and dissolved in 25 mL of water. For the pH determination, the honey solution was measured using a PHM 210 Standard pH Meter (MeterLab, Radiometer Copenhagen, Laan van Westenenk 50, Apeldoorn, The Netherlands), which was previously calibrated with standard pH 4 and pH 7 solutions. The same diluted solution was used for the electrical conductivity (EC) measurement using a conductometer (Conductimeter GLP 31 CRISON, Barcelona, Spain) calibrated with appropriate standard solutions. The results were expressed in mS/cm. The maximum EC for honey is 0.8 mS/cm according to Italian law (Directive 2001/110/EC), while for honeydew, multifloral/mixed, and chestnut honey, the EC values must be greater than 0.8 mS/cm.

**Figure 1:**
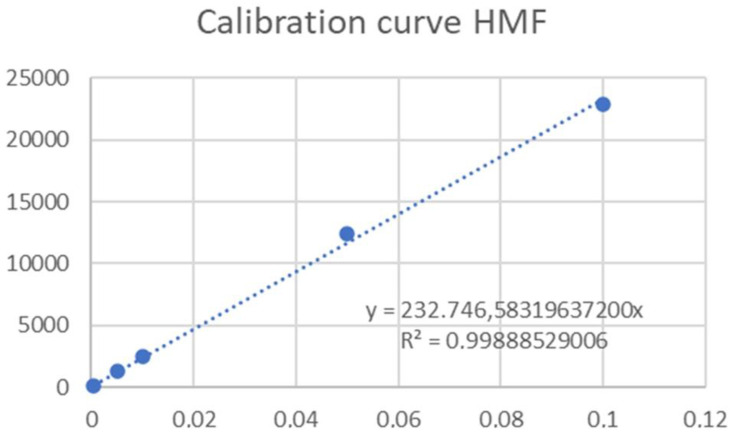
Calibration curve of HMF.

Hydroxymethylfurfural (HMF) was quantified following the HPLC method, which had been previously described in other studies with a few adjustments in accordance with Italian legislation guidelines [29,30], 5 g of each sample honey was diluted with ultrapure distilled water (1:5) and mixed. Then, within 12 h, samples were filtered on a 0.45 μm syringe filter, and 20 μL were injected into the HPLC system (Azura, Knauer, Berlin, Germany) coupled to a UV detector (Analytical UV Flow Cell Detector UVD 2.1S, Knauer, Berlin, Germany). The chromatographic column was Eurospher II 100-5 C18 150 × 4 mm, and the analysis conditions were: isocratic mobile phase, water–methanol 90:10 *v*/*v*; flow rate 0.6 mL/min; column temperature 30 °C. The detector wavelength was fixed at 285 nm, the identification of HMF was carried out by comparing the retention time of standard solution, and the quantification was performed using a calibration curve specific for each molecule (Figure 1). The calibration curve for HMF was made with five solutions at different concentrations (0.0005, 0.005, 0.01, 0.05, and 0.1 mg/mL. According to the law, the results were expressed in mg/kg, and the legal limit for HMF in commercial honey is 40 mg/kg.

The water content of the honey samples was determined with a handheld refractometer (HHTEC) with automatic temperature compensation. The samples were measured as-is, and the results are expressed as moisture content percent. The legal threshold for selling honey is 20%, but in competitions for premium/quality honey, the limit is usually lowered to 18%.

The brix degrees of the honey samples were measured with the same refractometer for moisture content measurement. Brix degrees represent the percentage of sugar content in honey by weight, with 1 Brix degree equivalent to 1 g of sucrose in 100 g of solution [31].

### 3.6. TAC, TPC, and Protein Content Determinations

Considering the paper of González-Ceballos et al. [32], and our previous paper on spirulina samples [28], hydrophilic extraction was chosen to determine TPC and TAC of the present honey samples and performed as follows: samples were extracted overnight in the dark in a ratio 1:25 (*w*/*v*) with phosphate-buffered saline (PBS). Then, the samples were centrifuged at 5000× *g* (ALC PK121R centrifuge; Bodanchimica s.r.l., Cagliari, Italy) for 10 min at 4 °C. The supernatants were collected and stored at −80 °C until further processing.

The TPC was determined using the Folin–Ciocalteu standard method as modified by Costantini et al. [33] and adapted for 96-well plates and an automatic reader (Infinite 2000, Tecan, Salzburg, Austria). Briefly, 30 μL of deionized water, was added to 10 μL of ethanolic extract, 10 μL of Folin–Ciocalteau reagent, and 200 μL of 30% Na_2_CO_3_. The absorbance of the mixture was measured at 725 nm on a plate reader (Infinite F200, TECAN, Männedorf, Switzerland). A gallic acid standard curve was prepared and the results were expressed as mg of gallic acid equivalents (GAE)/g of edible portion (EP) of the sample.

The total antioxidant capacity was assessed by two different methods: through ferric reducing antioxidant power (FRAP), and 2,2′-azino-bis (3-ethyl-benzothiazoline-6-sulfonic acid) (ABTS^•+^) radical scavenging activity assays as described as follows.

FRAP assay was performed using the method described by Benzie and Strain [34], which was adapted for 96-well plates and an automatic reader (Infinite 2000, Tecan, Salzburg, Austria). Briefly, 160 µL of FRAP assay solution (consisting of 20 mM ferric chloride solution, 10 mM TPTZ solution, and 0.3 M acetate buffer at pH 3.6) was prepared daily, mixed with 10 µL of the sample, standard, or blank, and dispensed into each well of a 96-well plate. The absorbance was measured at 595 nm at 37 °C after 30 min of incubation. The results were expressed as µmol Fe^2+^ equivalents/g EP.

The ABTS^•+^ radical scavenging activity was evaluated by the OxiSelectTM Trolox Equivalent Antioxidant Capacity (TEAC) Assay Kit (ABTS) (Cell Biolabs Inc., San Diego, CA, USA) following the manufacturer’s instructions. The absorbance was recorded at 405 nm in an automatic reader (Infinite 2000, Tecan, Salzburg, Austria). A standard curve for Trolox was prepared and the antioxidant capacity was expressed as μmol of Trolox equivalents (TE)/g EP.

Crude protein content (conversion factor, 6.25) was estimated using the Kjeldahl standard method AOAC 2001.11 [35].

### 3.7. Statistical Analysis

The mean and standard deviation (SD) of at least three biological replicates were calculated for all the analyzed data. Statistical analysis was performed with the XLSTAT 2023.2.1414 (Addinsoft SARL, Paris, France) software using Student *t*-test. Differences were considered significant at the *p* < 0.05.

## 4. Conclusions

In the present study, an evaluation of some parameters such as composition, chemical–physical properties, total phenolic and protein content and antioxidant capacity was carried out on a sample of wildflower honey before and after the addition of spirulina.

This enrichment led to significant variations in almost all of the measurements carried out, revealing how spirulina can influence the intrinsic characteristics of honey. In particular, although wildflower honey reinforced with spirulina was rich in bioactive components such as fatty acids and carboxylic acids, compared to the control, it showed a different sugar and VOC content and an increase in HMF and alcohol levels which could lead to a greater acidity of the honey. Furthermore, in honey with spirulina, electrical conductivity was increased, moisture content was reduced and no variation in pH was recorded. This study demonstrates how important chemical control of honey is in order to guarantee the quality of the product for a more conscious consumption with a view to functional foods.

## Figures and Tables

**Table 1:** Chemical volatile composition (percentage mean value ± standard deviation) of honey samples.

N°	Component ^1^	LRI ^2^	LRI ^3^	Wildflower Honey	Wildflower Honey + Spirulina
1	3-hexen-1-yne	623	625	8.0 ± 0.05	-
2	3-butyn-1-ol	662	660	0.5 ± 0.02	-
3	glutaraldehyde	890	895	0.7 ± 0.03	-
4	santolina alcohol	1038	1041	2.4 ± 0.08	-
5	linalool oxide	1060	1056	-	0.3 ± 0.02
6	isomyrcenol	1075	1072	2.7 ± 0.02	-
7	α-linalool	1091	1085	5.9 ± 0.03	-
8	nonanal	1107	1104	-	1.7 ± 0.03
9	hotrienol	1110	1114	36.5 ± 0.15	2.1 ± 0.04
10	nerol oxide	1140	1137	2.7 ± 0.02	-
11	nonanoic acid	1265	1260	7.2 ± 0.04	-
12	borneol acetate	1273	1270	4.2 ± 0.02	-
13	dehydro-ar-ionene	1338	1336	-	1.1 ± 0.03
14	(Z)-β-damascenone	1390	1382	-	0.2 ± 0.02
15	trans-β-ionone	1465	1460	-	2.3 ± 0.03
16	pentadecane	1510	1512	-	0.6 ± 0.02
17	lauric acid	1555	1561	2.3 ± 0.02	tr
18	hexadecane	1611	1612	-	0.8 ± 0.04
19	heptadecane	1708	1711	-	9.8 ± 0.05
20	palmitic acid	1945	1951	17.8 ± 0.11	24.3 ± 0.12
21	14-octadecenal	2000	2007	2.5 ± 0.02	-
22	oleic acid	2138	2141	5.0 ± 0.03	15.9 ± 0.09
23	elaidic acid	2145	2144	tr	40.7 ± 0.22
24	stearic acid	2168	2172	1.6 ± 0.04	tr
	SUM			100.0	99.8
	Monoterpenoids			5.1	2.8
	Monoterpenes			12.8	-
	Fatty acids			33.9	80.9
	Others			48.2	16.1

^1^ the components are reported according to their elution order on apolar column; ^2^ Linear Retention Indices measured on apolar column; ^3^ Linear Retention indices from the literature; tr: percentage mean values ˂ 0.1.

**Table 2:** Chemical composition (percentage values ± standard deviation) of derivatized extracts from dry honey samples.

N°	Components	Wildflower Honey	Wildflower Honey + Spirulina
**Carboxylic Acids**			
1	hydracrylic acid	-	0.3 ± 0.03
2	glycolic acid	tr	0.6 ± 0.03
3	benzoic acid	tr	0.2 ± 0.03
4	3-butenoic acid	tr	0.3 ± 0.03
5	3-methyl-2-furoic acid	-	2.4 ± 0.03
6	lactic acid	tr	-
**Alcohols**			
7	1-cyclopentanol	-	0.8 ± 0.03
8	2-ethoxyethanol	-	0.4 ± 0.03
9	3-octen-2-ol, (E)-	-	0.1 ± 0.03
10	phenol	tr	-
**Carbohydrates and Carbohydrate Derivatives**			
11	glycerol	tr	3.2 ± 0.05
12	ribitol	0.1 ± 0.01	0.3 ± 0.02
13	myo-inositol	10.6 ± 0.06	0.1 ± 0.00
14	D-gluconic acid	0.6 ± 0.03	-
15	D-talofuranose	0.2 ± 0.02	1.2 ± 0.03
16	D-tagatofuranose	0.4 ± 0.02	4.2 ± 0.05
17	D-ribofuranose	0.2 ± 0.02	15.7 ± 0.09
18	D-fructofuranose	28.3 ± 0.14	29.7 ± 0.11
19	D-erythrose	0.3 ± 0.02	-
20	D-fructose	21.1 ± 0.15	13.3 ± 0.08
21	D-glucose	6.3 ± 0.03	1.8 ± 0.04
22	psicofuranose	0.4 ± 0.02	1.0 ± 0.03
23	L-sorbofuranose	0.2 ± 0.02	4.4 ± 0.05
24	β-D-glucopyranose	25.6 ± 0.15	3.5 ± 0.03
25	β-D-xylofuranose	0.2 ± 0.02	-
26	2-deoxypentofuranose	-	3.7 ± 0.03
27	D-mannopyranose	0.1 ± 0.01	-
28	dihydroxyacetone	-	2.9 ± 0.02
29	levoglucosan	0.6 ± 0.02	-
30	turanose	1.5 ± 0.02	-
31	lactulose	1.3 ± 0.03	-
32	uridine	0.2 ± 0.02	4.5 ± 0.03
**Others**			
33	diethylene glycol	-	3.3 ± 0.03
34	furfuryl alcohol	-	0.4 ± 0.02
35	cadalene	0.5 ± 0.02	-
36	carvacrol	0.1 ± 0.01	-
37	trans-calamenene	0.1 ± 0.00	-
38	phloroglucinol	-	0.6 ± 0.02

**Table 3:** Physico-chemical data of honey samples (mean values ± SD).

Physico-Chemical Data	Wildflower Honey	Wildflower Honey + Spirulina
Brix%	81.77 ± 0.25	82.10 ± 0.40
Moisture content (%)	16.80 ± 0.20	15.90 * ± 0.10
EC (mS/cm)	0.74 ± 0.02	0.88 * ± 0.02
pH	4.47 ± 0.06	4.37 ± 0.06
HMF (mg/kg)	2.40 ± 0.10	8.13 * ± 0.47

* indicates significant differences between samples (*p* < 0.01).

**Table 4:** Total phenolic content (TPC), total antioxidant capacity (TAC), and protein content of honey samples.

	TPC mg GAE/g EP	FRAP µmol Fe^2+^E/g EP	ABTS^•+^ µmol TE/g EP	Protein Content g/100 g EP
Wildflower Honey	0.88 ± 0.03 ^b^	221.19 ± 11.67 ^a^	235.63 ± 6.39 ^b^	0.48 ± 0.03 ^b^
Wildflower Honey + spirulina	0.99 ± 0.02 ^a^	295.70 ± 46.89 ^a^	281.47 ± 36.18 ^a^	0.75 ± 0.07 ^a^

TPC: total phenolic content; GAE: gallic acid equivalent; EP: edible portion; FRAP: ferric reducing antioxidant power. ABTS^•+^: radical scavenging activity assays. TE: Trolox equivalent. Data are means ± standard deviation of three (n = 3) replicates. Means with different letters within a column are significantly different (*p* < 0.05).

## Data Availability

Data are available on request due to restrictions of privacy. The data presented in this study are available on request from the corresponding author.
